# Research in Iran: An Overview

**Published:** 2017

**Authors:** Shahin Akhondzadeh

Islamic Iran, on the Horizon of the 2025 Outlook Document, should obtain first regional rank in economic, social, scientific, and cultural domains. Country’s noticeable growth in scientific production and dynamic movement of Iranian scientists and specialists in scientific frontiers resulted in the country earning first rank in regional scientific production, leaving behind Turkey, in the year 2011, 14 years ahead of the Horizontal Outlook prediction. In the year 2016, Iran, with publication of 51187 articles in the Scopus database, acquired 16^th^ world-wide ranking. In the same year, citation to the Iranian articles was 28965, has achieved 18^th^ international ranking and 1st regional ranking. Overall, Iran, in the year 2016, has been responsible for an equivalent of 1.7% of the world’s total scientific production. Certainly, preserving Iran’s scientific growth trend is an important and fundamental subject and should consistently be brought to attention and consideration of government officials and policy makers in the research arena. There is no doubt; this support should include both basic and clinical studies ^1–3^.

Presence of national universities in international ranking institutions is considered one of the credible indicators for international scientific production for universities. Multiple international ranking institutions exist and university ranks in these institutions is considered to represent their qualitative and quantitative scientific production standing. One of the international ranking institutions, Essential Science Indicators (ESI), evaluates and ranks universities based on institution’s citation standing for the past ten years in the ISI database. From the sixty present national universities, currently thirteen national medical science universities are among world’s top universities in ESI. Before, this number was less and limited to a few universities. Among the top 500 universities in the world, just Tehran University of Medical Sciences is present.

Scientific production of Islamic Iran’s scientists holds the needed potential and wealth to be the world’s reference in science and knowledge. Based on this and by following guidelines of the supreme leader of the Islamic revolution, preservation, persistence, and reinforcement of the scientific production debate has unavoidable urgency and priority in academic and scientific circles and gatherings. Therefore, we must support innovative and technological institutes more than before.
